# Supplementary figures and images for: Modeling influenza seasonality in the tropics and subtropics
Source: PLoS Comput Biol. 2021 Jun 9;17(6):e1009050. doi: 10.1371/journal.pcbi.1009050 (PMC8216520; doi:10.1371/journal.pcbi.1009050)

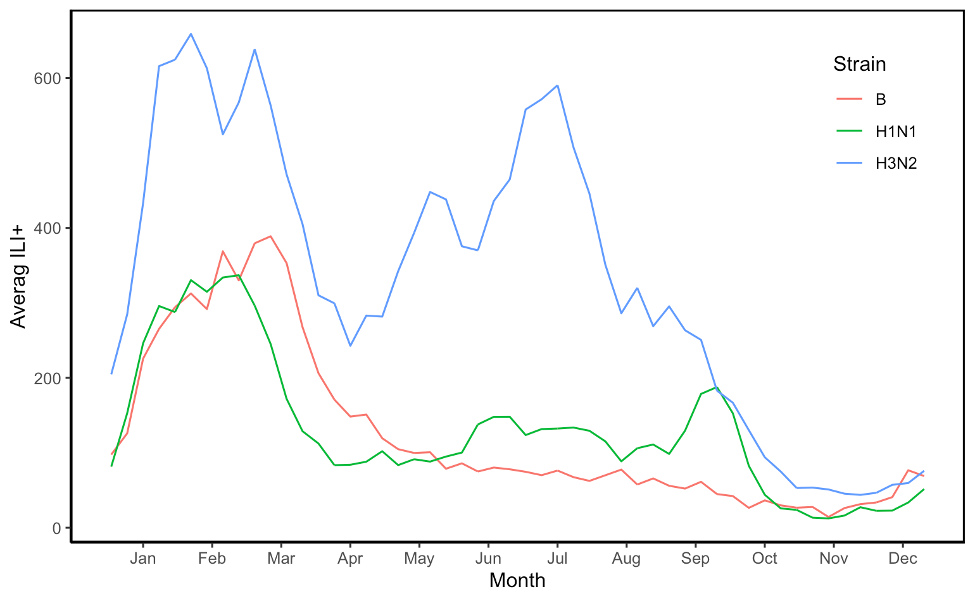

Supplement: S1 Fig — The mean ILI+ took average of the ILI+ observed in Hong Kong over 21 years (1998–2018). (TIF) [file pcbi.1009050.s001.tif]

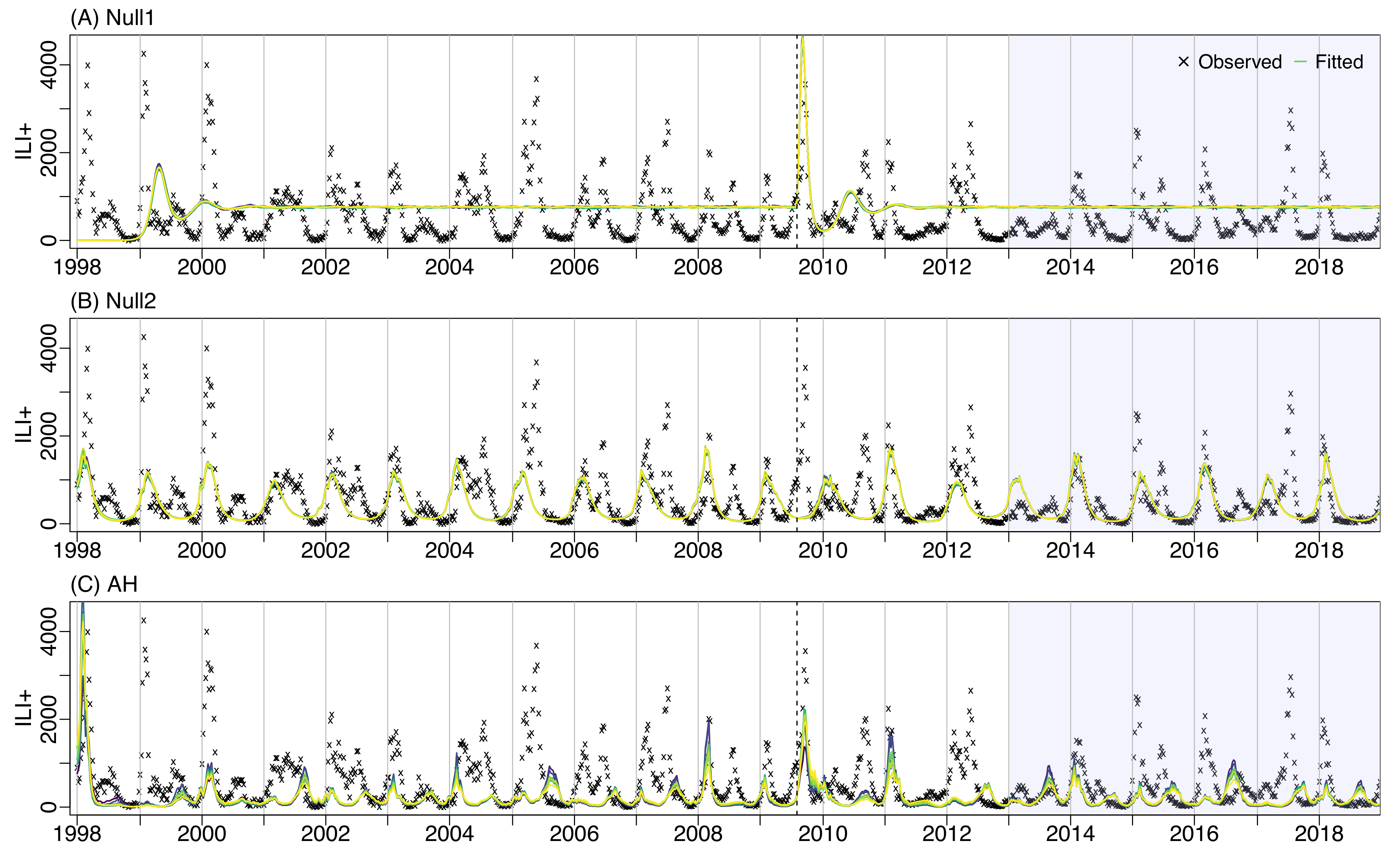

Supplement: S2 Fig — Top 10 model fits for Null1 (A), Null2 (B), and AH (C) model. Black crosses show observed ILI+; the colored lines run through the crosses show the top 10 model estimates. The vertical dash line indicates the onset of the 2009 pandemic. The shaded regions indicate testing years (2013–2018); and the rest are the training years. (TIF) [file pcbi.1009050.s002.tif]

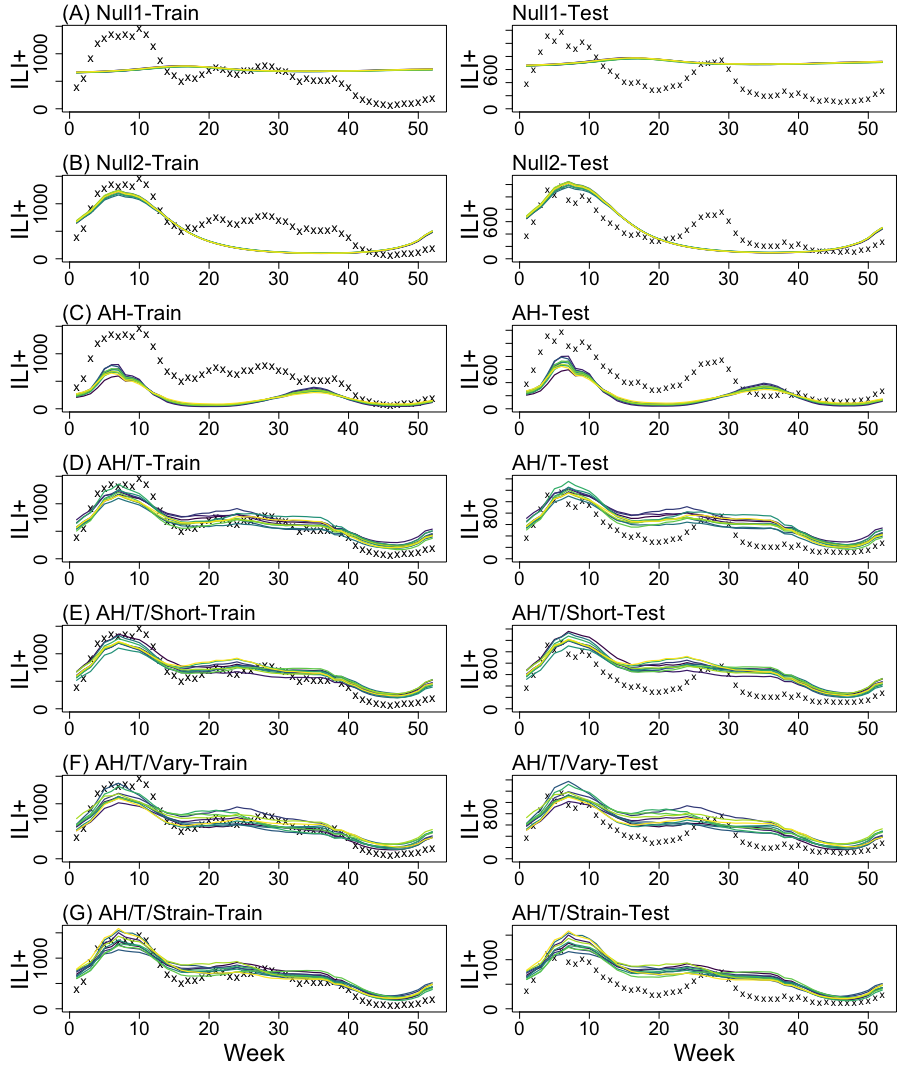

Supplement: S3 Fig — Top 10 model fits for the observed seasonality (averaged over training or testing years) for the seven models: Null1 (A), Null2 (B), AH (C), AH/T (D), AH/T/Short(E) AH/T/Vary (F) and AH/T/Strain (G). Black crosses show observed averaged ILI+ over training or testing years; colored lines run through the crosses show the top 10 model estimates. Left panels show model fits for the training period and the right panels show model fits for the testing period. (TIF) [file pcbi.1009050.s003.tif]

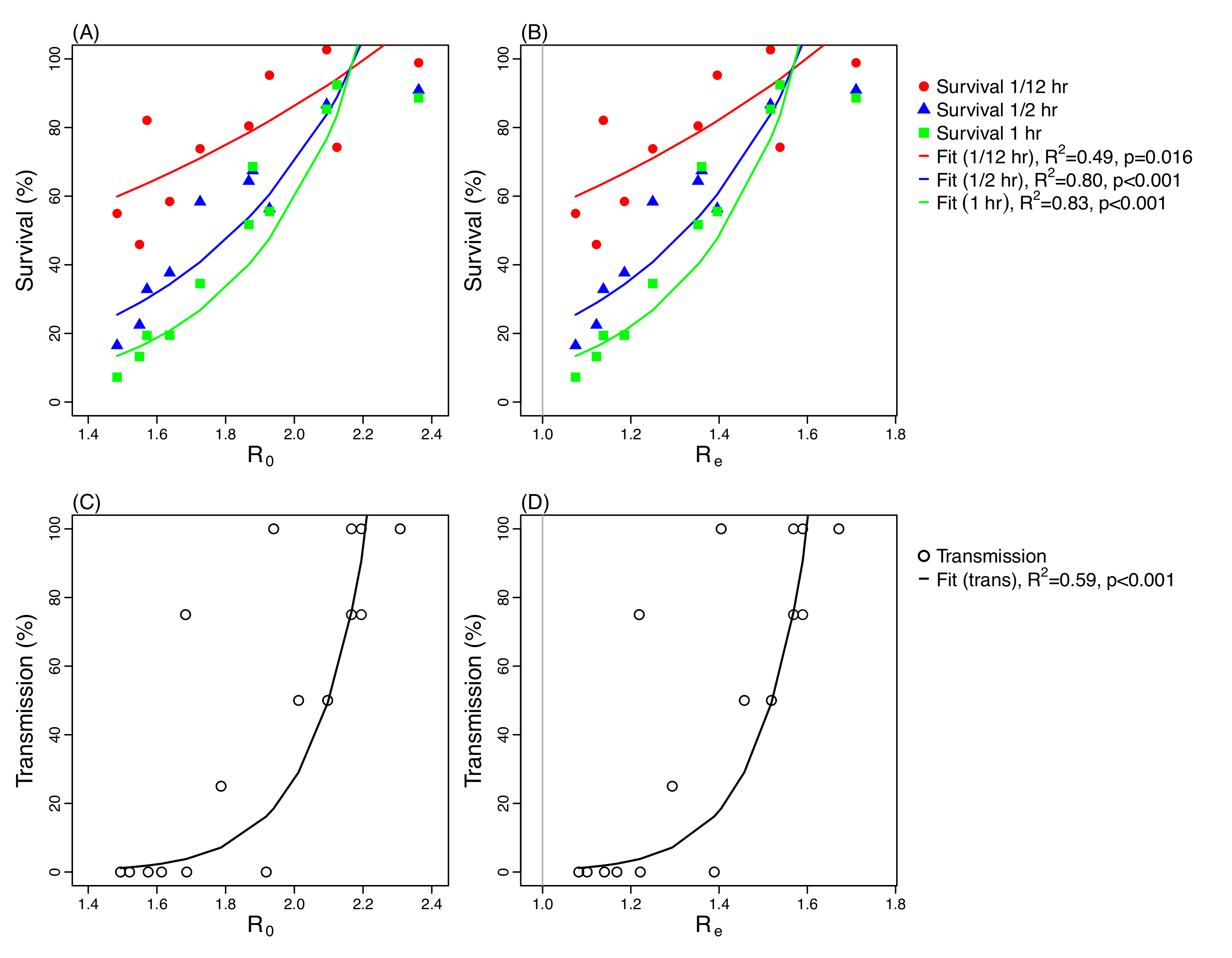

Supplement: S4 Fig — Same as in Fig 5 but using the Null 2 model instead. (TIF) [file pcbi.1009050.s004.tif]

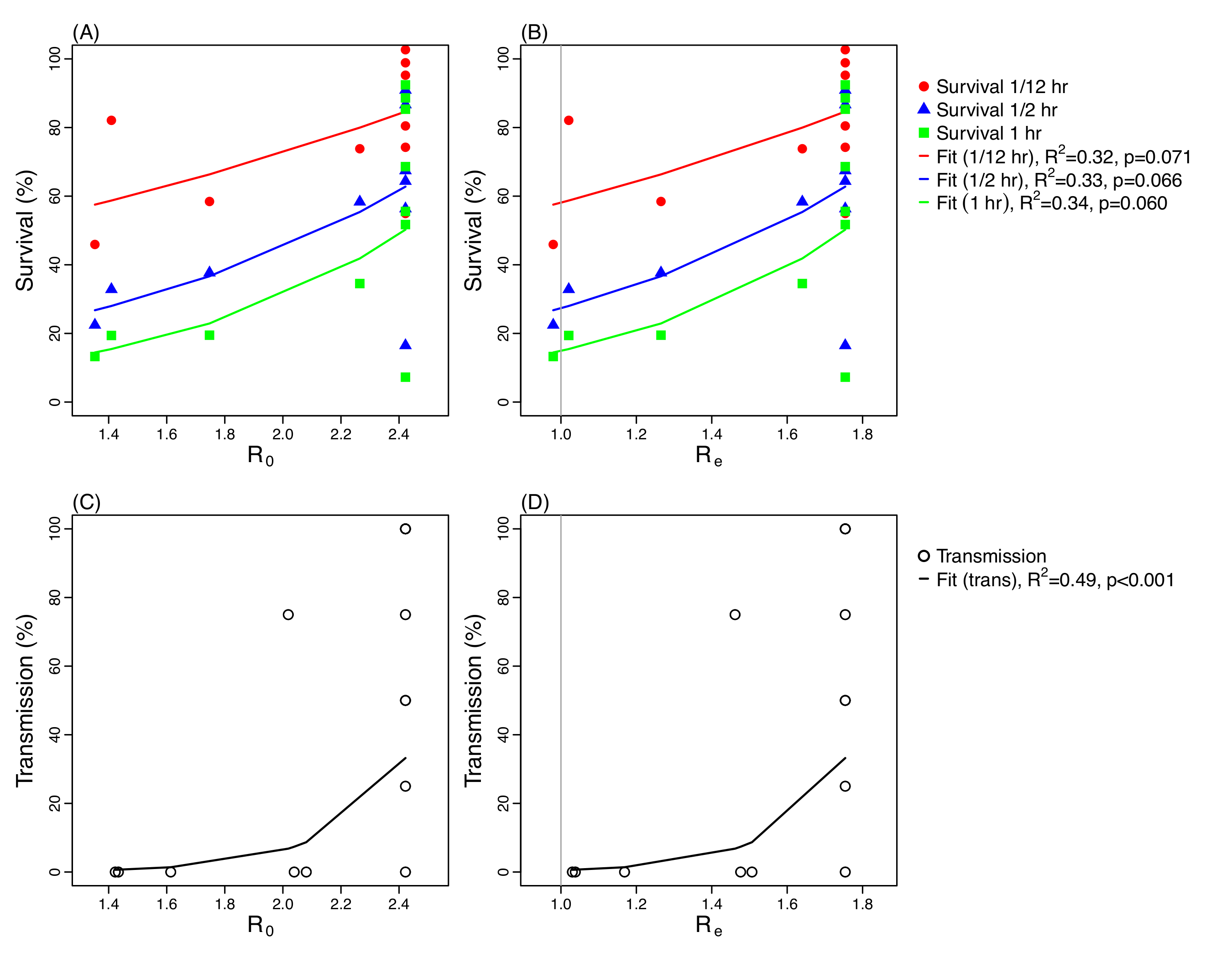

Supplement: S5 Fig — Same as in Fig 5 but using the AH model instead. (TIF) [file pcbi.1009050.s005.tif]

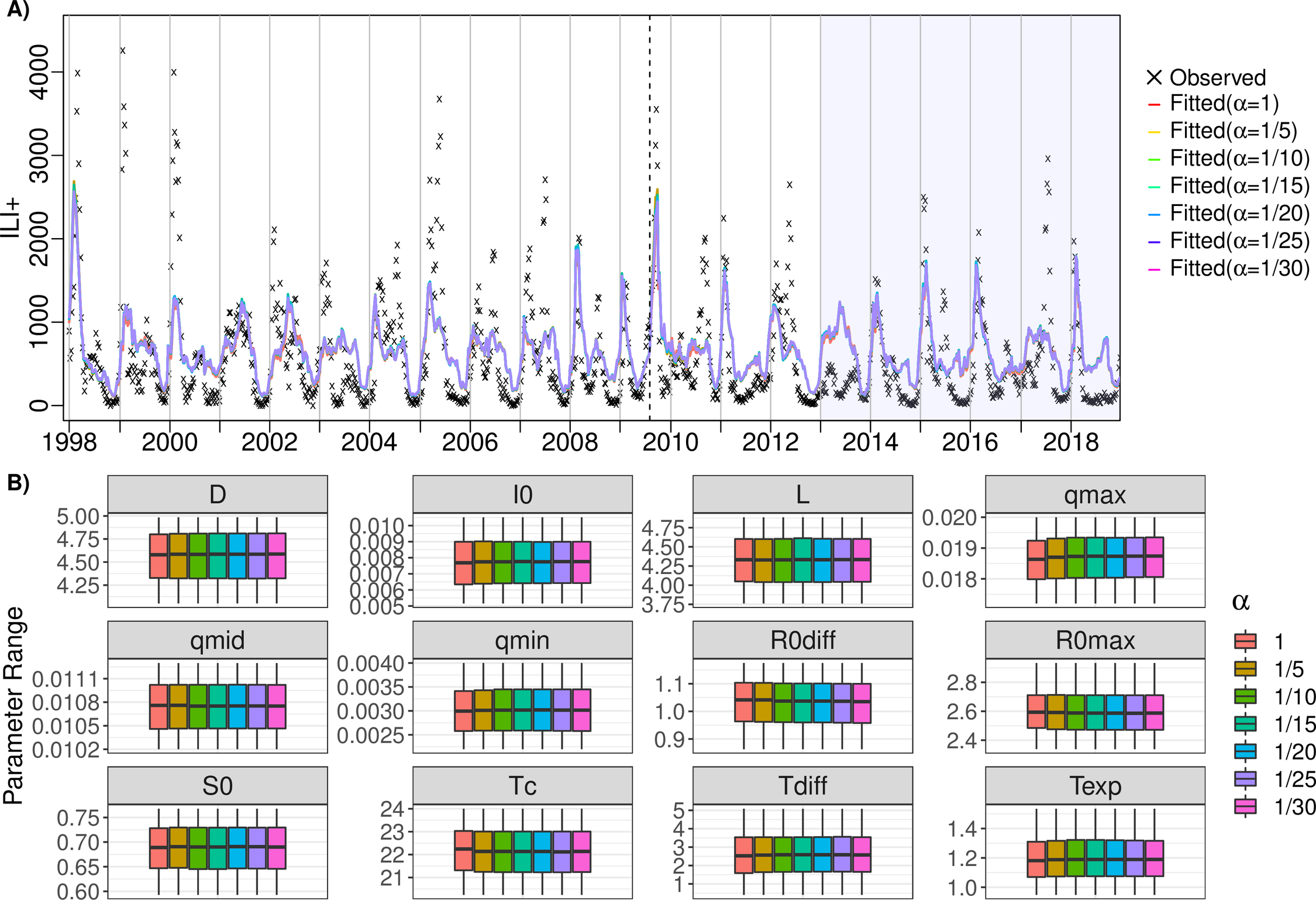

Supplement: S6 Fig — To test the model sensitivity to the value of ⍺, we ran the AH/T model along with the SIRS model using five different values (ranging from 1/30 to 1 as specified in the legend), separately. We optimized the model for each setting per the same procedure as described in the main text to estimate other parameters and variables. (TIF) [file pcbi.1009050.s006.tif]
