# Supplementary material for: Modeling influenza seasonality in the tropics and subtropics
Source: PLoS Comput Biol. 2021 Jun 9;17(6):e1009050. doi: 10.1371/journal.pcbi.1009050 (PMC8216520; doi:10.1371/journal.pcbi.1009050)
Supplement: S1 Text. Supplementary document includes 1) Preliminary data processing; 2) Simulation methods and Modeling detail; 3) Additional modeling results — (DOCX) [file pcbi.1009050.s009.docx]

**Supporting Information**

**Modeling influenza seasonality in tropics and subtropics**

Haokun Yuan, Sarah C. Kramer, Eric H. Y. Lau, Benjamin J. Cowling, Wan Yang

This supplemental document includes 1) Preliminary data processing; 2) Simulation methods and modeling details; and 3) Results testing an additional climate forcing model combining the relationship of influenza transmission with AH as in temperate climate (Null2 model) and the relationship with temperature as in our AH/T model.

**1. Preliminary data processing**

1.1 Calculation of absolute humidity [1]

First, we calculated saturation vapor pressure as:

|  | $e_{s}\left( T \right)=e_{s}\left( T_{0} \right)\times e^{\frac{L}{R_{v}}\left( \frac{1}{T_{0}}-\frac{1}{T} \right)}$ | [S1] |
| --- | --- | --- |

where *e_s_(T)* is the saturation vapor pressure at temperature *T* (in K), *e_s_(T_0_)* is the saturation vapor pressure at 273.15 K, *L* is the latent heat of evaporation for water, *R_v_* is the gas constant for water vapor. We then computed vapor pressure at each time point as:

|  | $e=e_{s}\left( T \right)\left( \frac{RH}{100} \right)$ | [S2] |
| --- | --- | --- |

where *RH* represents relative humidity. We calculated the mixing ratio, *mr*, as:

|  | $mr=\frac{R_{d}}{R_{v}}\left( \frac{e}{p_{0}-e} \right)$ | [S3] |
| --- | --- | --- |

where *R_d_* is the gas constant for dry air and *p_0_* is the atmospheric pressure at sea level. Finally, absolute humidity was calculated as:

|  | $AH=\frac{mr}{1+mr}$ | [S4] |
| --- | --- | --- |
|  |  |  |

**2. Simulation methods and modeling details**

2.1. Stochastic Model Runs

Similar to the climate forcing model used to model influenza in temperate region by Shaman et al. [2], we constructed a stochastic Markov chain, where stochastic is introduced by the transition between states. The number of individuals moving from one state to another (i.e. susceptible to infected, infected to recovered) is random draw from a Poisson distribution with a rate determined by Eqn. 1.

2.2. Climate forcing model for temperate regions (Null2)

The climate forcing model for temperate regions, first introduced by Shaman et al [2], assumes influenza transmission decreases monotonically with increasing absolute humidity. This relationship is modeled as:

|  | $R_{0}\left( t \right)=exp(a\times q\left( t \right)+b)+R_{0min}$ | [S5] |
| --- | --- | --- |

Where $a=-180,$ $b=\log\left( R_{0max}-R_{0min} \right), R_{0max}$ is the maximum daily basic reproductive number, while $R_{0min}$ is the minimum daily basic reproductive number.

2.3. Climate forcing model for (sub)tropical regions (AH/T, AH/T/Short, AH/T/Vary, AH/T/Strain)

Absolute humidity alone was proposed to have a bimodal effect on influenza transmission. And the formula can be written as follow:

|  | $R_{0}\left( t \right)=aq^{2}\left( t \right)+bq\left( t \right)+c$ | [S6] |
| --- | --- | --- |

Values of *a*, *b*, and *c* are defined as:

|  | $\left\{ \begin{matrix} a=\frac{-b}{q_{max}+q_{min}} \\ b=\frac{\left( R_{0_{max}}-\left( R_{0_{max}}-R_{0_{diff}} \right) \right)\left( q_{max}+q_{min} \right)}{\left( q_{max}-q_{mid} \right)\left( q_{min}-q_{mid} \right)} \\ c=\left( R_{0_{max}}-R_{0_{diff}} \right)-aq_{mid}^{2}-bq_{mid} \end{matrix} \right.$ | [S7] |
| --- | --- | --- |

Given equation S6, the derivation of equation S7 is shown as below:

$$\begin{matrix} R_{0_{max}}=aq_{min}^{2}+bq_{min}+c (1) \\ R_{0_{max}}=aq_{max}^{2}+bq_{max}+c (2) \\ R_{0_{min}}=aq_{mid}^{2}+bq_{mid}+c (3) \end{matrix}$$

Subtracting (1) from (2), we can get:

$$\begin{matrix} a\left( q_{max}^{2}-q_{min}^{2} \right)+ b(q_{\max}-q_{\min}) & = & 0 \end{matrix}$$

Rearrange the equation:

$$a=-\frac{b}{q_{max}+q_{min}}$$

Moreover, by subtracting (3) from (2), we can get:

$$R_{0_{\max}}-R_{0_{\min}}=a\left( q_{max}^{2}-q_{mid}^{2} \right)+ b\left( q_{max}-q_{mid} \right)$$

Plug in $a$:

$$R_{0_{\max}}-R_{0_{\min}}=b\left[ \left( q_{max}-q_{mid} \right)-\frac{q_{max}^{2}-q_{mid}^{2}}{q_{max}+q_{min}} \right]$$

Move $b$ to the left-hand side and rearrange:

$$b=\frac{\left( R_{0_{\max}}-\left( R_{0_{\max}}-R_{0_{\mathrm{diff}}} \right) \right)(q_{max}+q_{min})}{(q_{max}-q_{mid})(q_{min}-q_{mid})}$$

Plug $a$ and $b$ into Eqn. S7:

$c=\left( R_{0_{max}}-R_{0_{diff}} \right)-aq_{mid}^{2}-bq_{mid}$

**3. Null2/T model**

The Null2 (temperate climate) model assumes *R_0_* increases with decreasing humidity (see Eqn. S5 above and note that *a* = -180). Similarly, *R_0_* increases with decreasing temperature based on laboratory studies that showed influenza viruses survive longer under colder temperatures (see refs. 33, 35, 39 of the main text). Combining these two effects would make influenza transmission in the humid, hot summer in subtropical and tropical climates (e.g. in Hong Kong) more unlikely. As such, we did not include such a model in our analysis in the main text. However, to test this formally, here we present simulation results using such a Null2/T model – i.e., combining the temperature response as in the AH/T model (Eqn. 3; main text) and the temperate climate specific humidity model (Eqn. S5). Specifically, to include the impact of temperature, we modified Eqn. S5 for *R_0_* as:

|  | $R_{0}\left( t \right)=exp(a\times q\left( t \right)+b){[\frac{T_{c}}{T\left( t \right)}]}^{T_{exp}}+R_{0min}$ | [S8] |
| --- | --- | --- |

Note here the additional temperature term ${[\frac{T_{c}}{T\left( t \right)}]}^{T_{exp}}$is the same as in Eqn. 3. Specifically, *T(t)* is the temperature at time *t* and *T_c_* is the cutoff temperature. When *T(t)* is below *T_c_*, lower temperatures are able to further increase *R_0_*, whereas temperatures above *T_c_* inhibit influenza transmission.

Following the same optimization procedure as for other models, we found that the Null2/T model did not improve the model fit. As shown in S2 Table, the Null2/T model ranked slightly worse than the Null2 model and of all models, it was inferior in comparison with models incorporating the bimodal relationship between influenza transmission and humidity.

**Reference:**

1. Wallace J, Hobbs P. *Atmospheric Science: An Introductory survey.* 2nd Edition ed. New York: Academic Press; 2006. 504 p.

2. Shaman J, Pitzer VE, Viboud C, Grenfell BT, Lipsitch M. *Absolute humidity and the seasonal onset of influenza in the continental United States.* PLoS Biology. 2010;8(2).
